# Modulation of Cellular Hsp72 Levels in Undifferentiated and Neuron-Like SH-SY5Y Cells Determines Resistance to Staurosporine-Induced Apoptosis

**DOI:** 10.1371/journal.pone.0024473

**Published:** 2011-09-06

**Authors:** Lesley Cheng, Danielle J. Smith, Robin L. Anderson, Phillip Nagley

**Affiliations:** 1 Department of Biochemistry and Molecular Biology, Monash University, Clayton, Victoria, Australia; 2 Australian Research Council Centre of Excellence in Structural and Functional Microbial Genomics, Monash University, Clayton, Victoria, Australia; 3 Peter MacCallum Cancer Centre, Melbourne, Victoria, Australia; 4 Department of Pathology, The University of Melbourne, Parkville, Victoria, Australia; University of Turin, Italy

## Abstract

Increased expression of Hsp72 accompanies differentiation of human neuroblastoma SH-SY5Y cells to neuron-like cells. By modulating cellular levels of Hsp72, we demonstrate here its anti-apoptotic activity both in undifferentiated and neuron-like cells. Thermal preconditioning (43°C for 30 min) induced Hsp72, leading to cellular protection against apoptosis induced by a subsequent treatment with staurosporine. Preconditioned staurosporine-treated cells displayed decreased Bax recruitment to mitochondria and subsequent activation, as well as reduced cytochrome *c* redistribution from mitochondria. The data are consistent with Hsp72 blocking apoptosis upstream of Bax recruitment to mitochondria. Neuron-like cells (with elevated Hsp72) were more resistant to staurosporine by all measured indices of apoptotic signaling. Use of stable transfectants ectopically expressing moderately elevated levels of Hsp72 revealed that such cells in the undifferentiated state showed enhanced resistance to staurosporine-induced apoptosis, which was even more robust after differentiation to neuron-like cells. Overall, the protective effects of differentiation, thermal preconditioning and ectopic Hsp72 expression were additive. The strong inverse correlation between cellular Hsp72 levels and susceptibility to apoptosis support the notion that Hsp72 acts as a significant neuroprotective factor, enabling post-mitotic neurons to withstand potentially lethal stress that induces apoptosis.

## Introduction

Apoptosis in neurons contributes to pathological conditions, such as the acute brain injury that occurs in stroke, or the chronic injury in neurodegenerative disorders [1]. In particular, the mitochondrial pathway of apoptosis can be elicited by cellular stresses, including DNA damage or loss of survival-inducing intracellular or extracellular signaling pathways [2], [3]. In response to cellular stresses, Bax is recruited to the mitochondria where it is activated, leading to redistribution of intermembrane space proteins such as cytochrome *c* (cyt c) from the mitochondria to the cytosol [3]. Cyt c in the cytosol associates with Apaf-1 to promote assembly of Apaf-1 into the multi-protein apoptosome structure. The apoptosome recruits and activates procaspase-9, which then cleaves other procaspases such as procaspase-3, thereby initiating a caspase cascade, cleaving key cellular substrates that generate apoptotic changes in the cell, including characteristic changes in nuclear morphology [2].

The tendency of cells to undergo apoptosis can be modulated by intracellular factors, some of which are induced as a result of mild stress. For example, Hsp72 is often induced during cellular stress to repair damage, maintain cellular homeostasis and facilitate the recovery of cells from otherwise lethal stimuli [4], [5]. Thus, Hsp72 is upregulated in injured and damaged areas of the brain during a variety of external stresses such as hyperthermia, stroke, ischemia and acute brain injury [6]. At a cellular level, Hsp72 is upregulated in neuronal cells under thermal preconditioning, a non-lethal thermal stress that protects cells from a subsequent, otherwise lethal, cellular insult [7], [8], [9], [10], [11].

Recent evidence supports the notion that Hsp72 is able to protect cells from lethal stresses by its ability to specifically block apoptotic pathways in cells upstream of mitochondria [12], [13], despite earlier claims to the contrary [14], [15]. We have shown that increased expression of Hsp72 accompanies the differentiation of human neuroblastoma SH-SY5Y cells, driven by retinoic acid and brain derived neurotrophic factor, to neuron-like cells [11]. Using hyperthermic stress as a cellular insult, we demonstrated that Hsp72 has a major role in the enhanced hyperthermic resistance acquired during neuronal differentiation of SH-SY5Y cells [11]. Recognizing that hyperthermic cell death is often manifested as apoptotic death [16], [17], although severe or prolonged heat treatments induce necrosis [11], [18], we reasoned that it would be important to apply a specific apoptosis inducer to determine in more detail the mechanism by which Hsp72 blocks death in this neuronal system. Accordingly, we selected the kinase inhibitor staurosporine [19], in light of its well-known ability to induce apoptosis [20], [21], [22].

In this report, we used SH-SY5Y cells to study the protective effects of neuronal differentiation, induction of thermotolerance (through thermal preconditioning), and ectopic expression of Hsp72, on apoptotic signaling induced by STS. Our results establish a strong relationship between the resistance to STS-induced apoptosis in SH-SY5Y cells and the level of Hsp72. The data indicate that the protective effects of Hsp72 lie upstream of mitochondrial engagement in apoptotic signaling.

## Results

### Apoptotic nuclear fragmentation is reduced in thermally preconditioned SH-SY5Y cells in response to STS treatment

**Figure 1:**
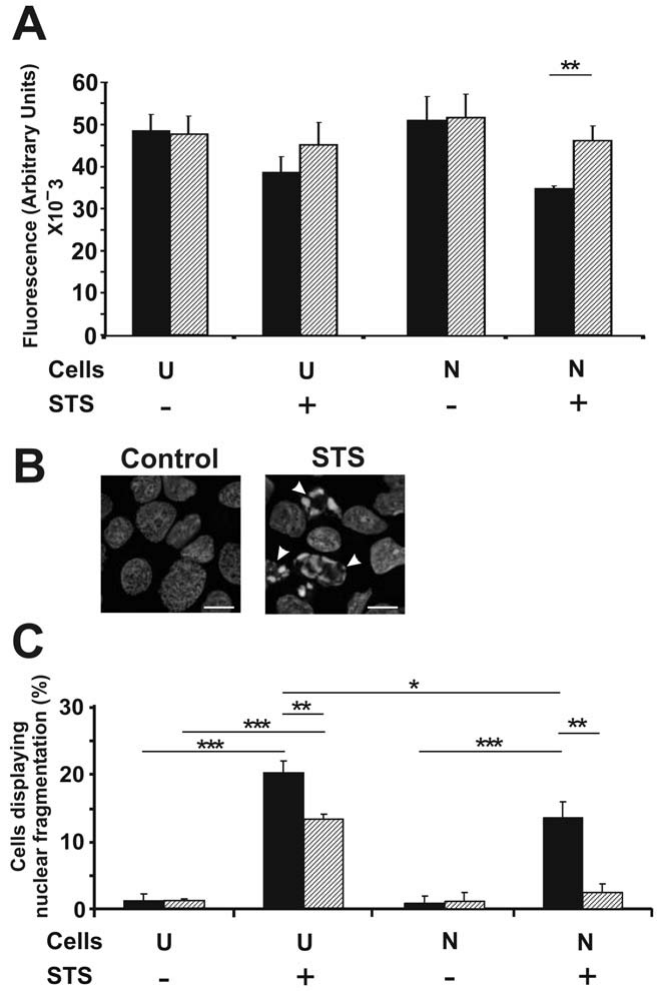
Thermal preconditioning protects SH-SY5Y cells from cell death and nuclear fragmentation induced by STS. (A) SH-SY5Y cells, undifferentiated (U) or neuron-like (N), were either kept at 37°C (black bars) or thermally preconditioned (striped bars) prior to treatment for 12 h with STS (50 nM). Cells were harvested and a cell viability assay (Cell Titer-Blue) was carried out. (B) Images of DAPI-stained undifferentiated SH-SY5Y cells. Arrows indicate fragmented apoptotic nuclei. Similar images were obtained with neuron-like SH-SY5Y cells (data not shown). Bar = 10 µm. (C) Nuclear fragmentation was quantified in SH-SY5Y cells, undifferentiated (U) or neuron-like (N), each treated with 50 nM STS for 12 h with (striped bars) or without (black bars) thermal preconditioning. For data in panels A and C, at least 300 cells were scored for each of three independent experiments; error bars indicate standard deviation. *p<0.05; **p<0.01; ***p<0.0001.

Applying a Cell Titer-Blue cell viability assay, undifferentiated SH-SY5Y cells subjected to thermal preconditioning showed modest protection against death induced by 50 nM STS for 12 h, whereas neuron-like SH-SY5Y cells similarly treated with 50 nM STS show significantly greater cell survival when thermally preconditioned (*p*<0.01) (Figure 1A). Higher concentrations of STS tend to kill cells regardless of differentiation or thermal preconditioning (data not shown).

**Figure 2:**
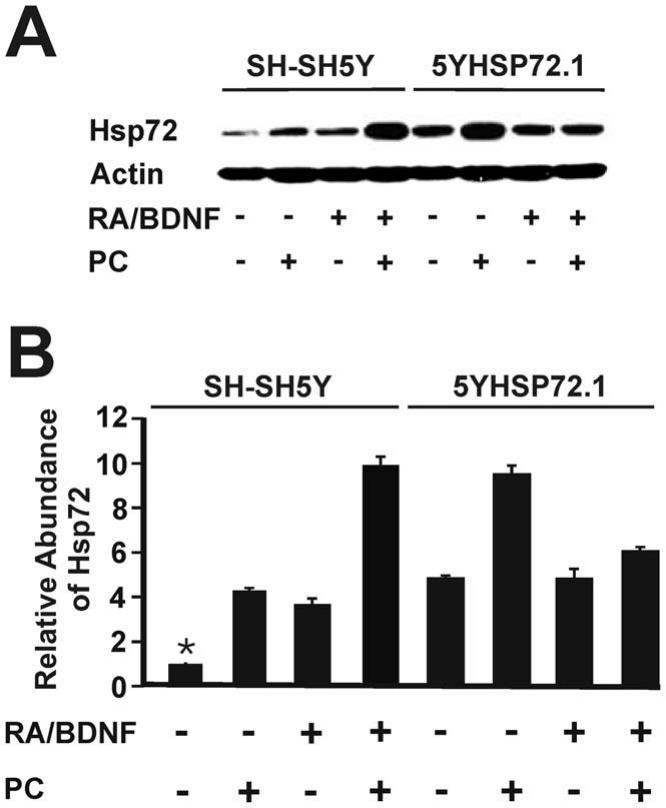
Hsp72 expression levels of SH-SY5Y and 5YHSP72.1 cells. (A) SH-SY5Y and 5YHSP72.1 cells were cultured in the undifferentiated form (RA/BDNF −) or differentiated with RA and BDNF (RA/BDNF +). Cells were then either kept at 37°C or thermally preconditioned (PC) at 43°C for 30 min, followed by a recovery period of 8 h at 37°C before harvesting. Cell lysates were collected and subjected to Western immunoblotting with anti-Hsp72 and actin antibodies. (B) Levels of Hsp72 induction were quantified by densitometric analysis of the type of image shown in panel A and in ref. [11]. Data in Panel B were adapted from those shown in ref. [11], with permission from the publisher. * Fold induction values from all densitometric analyses here were normalised to the levels of Hsp72 in undifferentiated SH-SY5Y cells. All results are from three independent experiments. Error bars indicate standard deviation.

Induction of apoptosis in SH-SY5Y cells by STS was indicated by characteristic changes in nuclear morphology (Figure 1B). In contrast to nuclei in untreated cells, chromatin in apoptotic nuclei is typically condensed and fragmented. Following a 12-h exposure to STS (50 nM), undifferentiated SH-SY5Y cells showed about 20% apoptotic cells (p<0.0001), while neuron-like cells showed a significantly smaller induction of apoptosis, about 15% (*p*<0.001) (Figure 1C). As observed in previous studies in our laboratory [11], SH-SY5Y cells differentiated with RA and BDNF were found to express increased levels of Hsp72 compared to undifferentiated SH-SY5Y cells (Figure 2A and B, lanes 1 and 3). The resistance to STS treatment in neuron-like SH-SY5Y cells is consistent with the upregulation of Hsp72 upon differentiation. Acquisition of such resistance to STS-induced death, comparing neuron-like and undifferentiated SH-SY5Y cells, is not reflected in the Cell Titer-Blue assays (Figure 1A). This is because of the relatively low sensitivity of this gross metabolic assessment, nonetheless useful to determine an appropriate concentration of STS for the present studies.

In the case of SH-SY5Y cells that were thermally preconditioned to upregulate Hsp72 and subsequently treated with STS, both undifferentiated and neuron-like SH-SY5Y are significantly protected from STS as measured by nuclear fragmentation (p<0.01, in each case) (Figure 1C). Thus, in preconditioned undifferentiated cells, preconditioning reduced apoptosis to 15%, while neuron-like SH-SY5Y cells are almost completely protected. These findings are in accord with the upregulation of Hsp72 by thermal preconditioning, in each case elevating the levels of Hsp72 above the base level in the respective cells (Figure 2).

### The redistribution of cytochrome c from mitochondria in response to STS is reduced in thermally preconditioned SH-SY5Y cells

**Figure 3:**
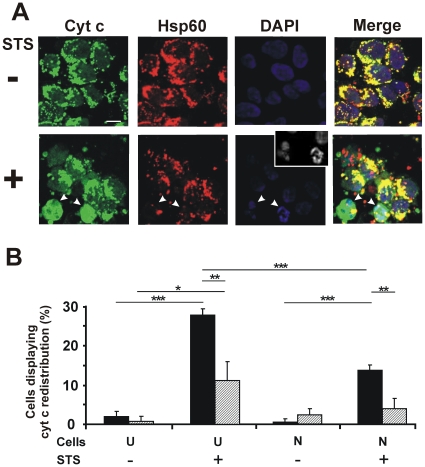
Thermal preconditioning blocks STS-induced redistribution of cyt c from the mitochondria. (A) Images of undifferentiated SH-SY5Y cells treated without (−) or with (+) STS (50 nM for 12 h). Cells were immunostained for cyt c (Alexa 488 labeled, green channel) and Hsp60 (Alexa 568 labeled, red channel). The same fields stained with DAPI are shown (inset shows fragmented nuclei in grayscale for clearer visibility). Merge of first three images is shown in the far right panels. Similar images were obtained for neuron-like SH-SY5Y cells (data not shown). Bar = 10 µm. (B) Quantified data for cells displaying cyt c redistribution from the mitochondria in undifferentiated (U) and neuron-like (N) cells before (black bars) and after (striped bars) thermal preconditioning. At least 300 cells were scored for each of three independent experiments; error bars indicate standard deviation. *p<0.05; **p<0.01; ***p<0.0001.

To establish more precisely where Hsp72 may act to effect the apoptotic block, steps in the mitochondrial pathway to apoptosis were examined. Subsequent to thermal preconditioning, undifferentiated and neuron-like SH-SY5Y cells were treated with STS. Cells were harvested, fixed and immunostained using mouse anti-cyt c antibody, and also exposed to DAPI (4′,6-diamidino-2-phenylindole) to stain the nuclei (Figure 3A). Images of control cells show that cyt c colocalizes with Hsp60 in mitochondria. After STS treatment, cyt c in many cells redistributes to the cytosol and often can be visualized to lie over the nuclear region [21], [23], the latter indicated by DAPI staining to be apoptotic in morphology (Figure 3A, inset). Hsp60 staining (red channel) reveals the residual location of mitochondria (typically pyknotic in these apoptotic cells), clustered around the nucleus [24].

Quantified data (Figure 3B) show that 28% of undifferentiated SH-SY5Y cells in the absence of thermal preconditioning have undergone cyt c redistribution in response to STS, with a markedly reduced proportion in neuron-like SH-SY5Y cells (14%, *p*<0.0001). Subsequent to thermal preconditioning, both undifferentiated and differentiated SH-SY5Y cells are protected to a significant degree (*p*<0.01) relative to their non-preconditioned counterparts. These results indicate that the putative apoptotic block effected by Hsp72 is upstream of cyt c release from mitochondria.

### The activation of Bax at the mitochondria and recruitment of Bax to mitochondria in response to STS are reduced in thermally preconditioned SH-SY5Y cells

Recruitment of Bax to mitochondria and its activation on the outer membrane are required for outer membrane permeabilization leading to cyt c redistribution. The conformationally active state of Bax in thermally preconditioned and STS-treated SH-SY5Y cells was investigated, using an antibody that specifically recognizes an epitope of Bax that is exposed only when it has been activated [25]. This was achieved by using CHAPS buffer for antibody permeation into fixed cells, as described previously [25].

**Figure 4:**
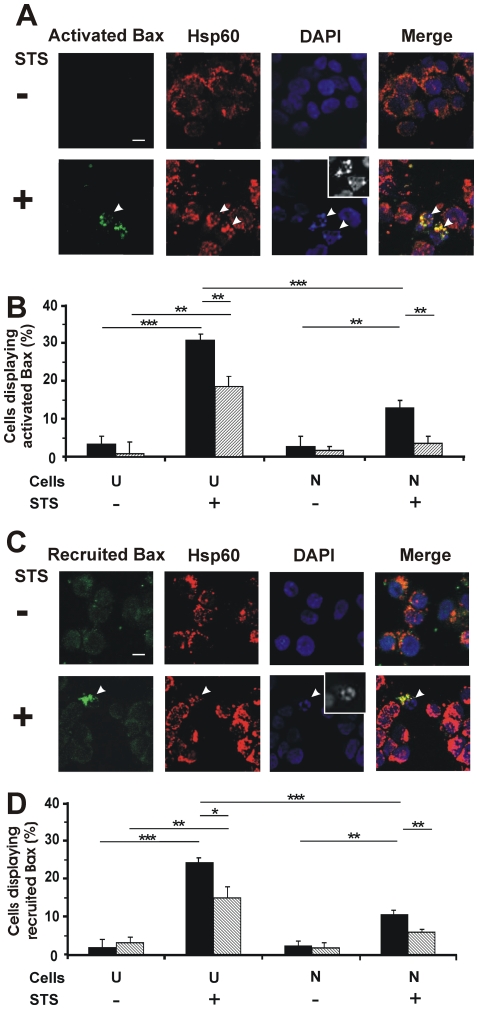
Thermal preconditioning blocks STS-induced Bax activation and Bax recruitment at mitochondria. (A) Images of undifferentiated SH-SY5Y cells before (−) or after (+) STS treatment (50 nM for 12 h). Cells were immunostained for activated Bax (Alexa 488 labeled, green channel) and Hsp60 (Alexa 568 labeled, red channel). The same fields stained with DAPI are shown (inset shows fragmented nuclei in grayscale for clearer visibility). Merge of first three images is shown in the far right panels. Similar images were obtained for neuron-like SH-SY5Y cells (data not shown). Bar = 10 µm. (B) Quantified data for cells displaying activated Bax at the mitochondria in undifferentiated (U) and neuron-like (N) cells. At least 300 cells were scored for each of three independent experiments; error bars indicate standard deviation. *p<0.05; **p<0.01; ***p<0.0001. (C) Images of undifferentiated SH-SY5Y cells treated without (−) or with (+) STS (50 nM for 12 h). Cells were immunostained for Bax distribution (Alexa 488 labeled, green channel) and Hsp60 (Alexa 568 labeled, red channel). Other indications are as for Panel A. (D) Quantified data for cells displaying Bax recruited to the mitochondria in undifferentiated (U) and neuron-like (N) cells. At least 300 cells were scored for each of three independent experiments; error bars indicate standard deviation. *p<0.05; **p<0.01; ***p<0.0001.

Undifferentiated SH-SY5Y control cells (Figure 4A, top row) fail to show activated Bax, as expected. Similar images were obtained for neuron-like SH-SH5Y cells (data not shown). These untreated cells clearly show many mitochondria by Hsp60 staining and display normal nuclei by DAPI staining. Amongst cells treated with STS (Figure 4A, bottom row) some show Bax activated at the mitochondria, the punctate pattern of activated Bax colocalizing with Hsp60. These cells also display apoptotic nuclei (inset).

Quantified data (Figure 4B) reveal a significantly reduced extent (*p*<0.001) of Bax activation (13%) occurring in neuron-like SH-SY5Y cells treated with STS compared to their undifferentiated counterparts (30%). With thermal preconditioning, undifferentiated and neuron-like SH-SY5Y cells are both protected against Bax activation. This protection is more extensive in neuron-like SH-SY5Y cells relative to their undifferentiated counterparts. As above, protective effects of differentiation and thermal preconditioning are additive.

In confirmation of this study on Bax activation, Bax recruitment to the mitochondria was monitored in thermally preconditioned SH-SY5Y cells treated with STS. Fixed cells were permeabilized with Triton-X-100 (as opposed to CHAPS buffer) and immunostained by rabbit anti-Bax antibodies, allowing the detection of both non-active and activated Bax.

In untreated cells (Figure 4C, top row), Bax shows diffuse staining throughout the cells, quite distinct from mitochondria (identified by Hsp60 staining). In contrast, some STS-treated cells have recruited Bax to mitochondria (Figure 4C, bottom row), the Bax staining colocalizing with that of Hsp60. In such cells, nuclei are typically apoptotic (inset).

Quantified data (Figure 4D) indicate reduced Bax recruitment (*p*<0.0001) occurring in neuron-like SH-SY5Y cells (11%) treated with STS, compared to their undifferentiated counterparts (25%), as for Bax activation (Figure 4B). Likewise, after thermal preconditioning, both undifferentiated and neuron-like SH-SY5Y cells show enhanced deficits in Bax recruitment, the latter more so. Collectively, these data on mitochondrial indicators of apoptosis clearly indicate that the apoptotic block by Hsp72 is upstream of Bax recruitment to mitochondria.

### Apoptotic signaling is blocked upstream of mitochondria in cells over-expressing Hsp72

To demonstrate more directly the requirement for Hsp72 in achieving protection against apoptotic signaling induced by STS, stably transfected 5YHSP72.1 cells with moderately elevated Hsp72 levels, within the physiological range (Figure 2A and B, Lane 5), were assessed. SH-SY5Y and 5YHSP72.1 cells were each treated with STS for 12 h and monitored for apoptotic markers, namely, nuclear fragmentation and Bax activation (the latter is more clearly visualized than Bax recruitment, cf. Figure 4A and C).

**Figure 5:**
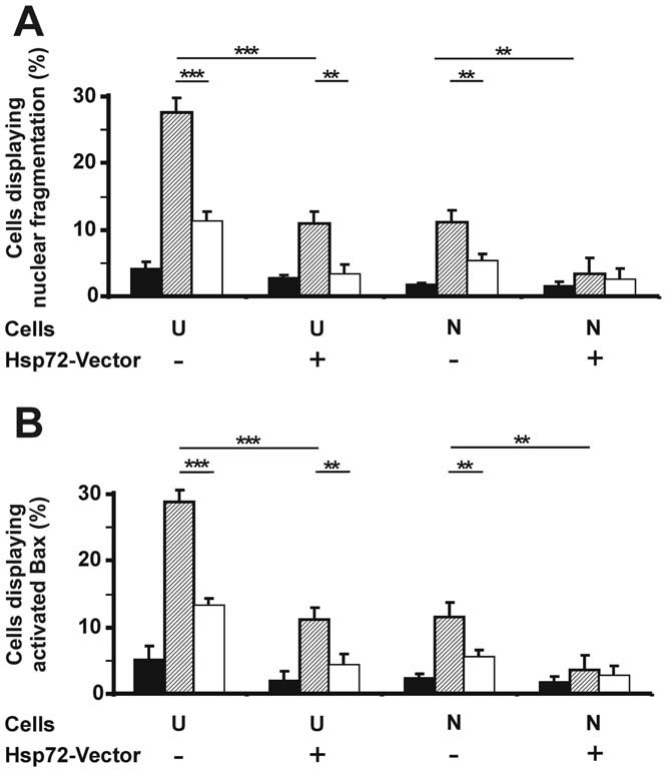
Ectopic expression of Hsp72 confers resistance to apoptosis induced by STS. SH-SY5Y cells (Hsp72-Vector −) were compared to 5YHSP72.1 cells (Hsp72-Vector +), in each case either undifferentiated (U) or neuron-like (N). Control cells (black bars) were kept at 37°C while other cells were treated with STS (50 nM for 12 h), either without (striped bars) or with prior thermal preconditioning (white bars). Quantified data are shown for nuclear fragmentation (A) and Bax activation (B). At least 300 cells were scored for each of three independent experiments; error bars indicate standard deviation. **p<0.01; ***p<0.0001.

Undifferentiated 5YHSP72.1 cells are protected against the apoptotic effects of STS treatment compared to undifferentiated SH-SY5Y cells, as assessed by both nuclear fragmentation (Figure 5A) and Bax activation (Figure 5B). Thus, whereas undifferentiated SH-SY5Y cells show about 25–30% of cells with either of these two apoptotic markers, after STS treatment, the stably transfected undifferentiated 5YHSP72.1 cells show significantly reduced extents of apoptosis (about 10% for each marker; *p*<0.001). Moreover, neuron-like 5YHSP72.1 cells showed an almost complete deficit in Bax activation and nuclear fragmentation when treated with STS (about 5% for each marker), similar to basal levels seen in untreated cells.

Thermally preconditioned undifferentiated 5YHSP72.1 cells displayed significant induction levels of Hsp72 (Figure 2A and B, Lane 6) and further suppressed STS-induced apoptosis, indicated by criteria of both nuclear fragmentation (Figure 5A) and Bax activation (Figure 5B) (*p*<0.01). Thermal preconditioning had little demonstrable further effect on neuron-like 5YHSP72.1 cells, as they already showed almost a complete deficit of Bax activation and nuclear fragmentation.

### Pharmacological inhibition of Hsp72 by KNK437 restores susceptibility of cells to STS-induced apoptosis

To strengthen the proposal that Hsp72 can suppress mitochondrial apoptotic signalling induced by STS, ablation of Hsp72 would be a desirable approach. However, since siRNA approaches to achieve knockdown of Hsp72 were unsuccessful (data not shown), pharmacological blockade of Hsp72 synthesis was applied by use of KNK437 (N-formyl-3,4-methylenedioxy-benzylidene-γ-butyrolactam). This compound was shown previously to abrogate the acquisition of thermotolerance in SH-SY5Y and 5YHSP72.1 cells [11].

**Figure 6:**
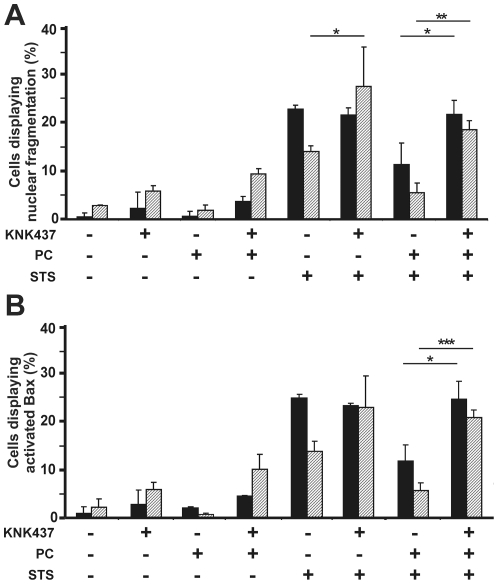
KNK437 abrogates the resistance to STS-induced apoptosis in SH-SY5Y and 5YHSP72.1 cells. SH-SY5Y cells (black bars) and 5YHSP72.1 (striped bars) were thermally preconditioned (PC) or not, 8 h before treatment with 50 nM STS for a further 12 h. Where indicated, KNK437 was added to undifferentiated SH-SY5Y cells (50 µM) or 5YHSP72.1 cells (75 µM) 1 h before thermal preconditioning. Quantified data are shown for nuclear fragmentation (A) and Bax activation (B). At least 300 cells were scored for each of three independent experiments; error bars indicate standard deviation. *p<0.05; **p<0.01; ***p<0.0001.

Undifferentiated SH-SY5Y and 5YHSP72.1 cells were exposed to KNK437 (50 µM and 75 µM, respectively) for 1 h prior to thermal preconditioning, followed by treatment with 50 nM STS for a further 12 h. The concentrations of KNK437 used here for both cell types were found previously to inhibit Hsp72 induction in thermally preconditioned cells to untreated control levels of Hsp72 [11]. Control experiments without either thermal preconditioning or STS but in the presence of KNK437, showed that by itself this drug did not cause significant toxicity in either undifferentiated SH-SY5Y or 5YHSP72.1 cells, as assessed by nuclear fragmentation (Figure 6A) or Bax activation (Figure 6B).

In STS-treated undifferentiated SH-SY5Y cells (not subjected to thermal preconditioning), the addition of KNK437 does not significantly alter the extent of apoptosis, relative to cells treated with STS alone (Figure 6A and 6B). However, 5YHSP72.1 cells (with no thermal preconditioning) treated with STS in the presence of KNK437 displayed a significant increase in nuclear fragmentation (*p*<0.05) and notable increases of Bax activation (*p* = 0.08) (about 25% for both markers) compared to 5YHSP72.1 cells treated with STS alone (about 15% for both markers). The results indicate that ongoing synthesis of Hsp72 driven by the vector in 5YHSP72.1 cells, which is blocked by KNK437, is needed for cellular protection against STS.

Significantly, in the cells exposed to thermal preconditioning, KNK437 completely abrogates the resistance to STS-induced apoptosis for both SH-SY5Y and 5YHSP72.1 cells. Thus, the apoptotic indices of nuclear fragmentation (Figure 6A) and Bax activation (Figure 6B) are both restored to the levels seen in control undifferentiated cells treated with STS. This loss of protection can be explained by the inhibition of Hsp72 synthesis effected by KNK437, with consequent loss of protection of cells against apoptotic death induced by STS.

### Inverse correlation between cellular Hsp72 levels and susceptibility to apoptosis

Based on the results described above for cellular protection against STS mediated by neuronal cell differentiation and thermal preconditioning, it is possible to directly correlate such protection with cellular Hsp72 levels in Figure 2. The data integrating these two factors are collated in Table 1, segregated into undifferentiated and neuron-like cells, for convenience of presentation. Data for KNK437-inhibited apoptotic signalling are not included in Table 1, as the data are essentially redundant due to the demonstrated pharmacological block on both cell death (Figure 6) and Hsp72 induction [11] in the relevant undifferentiated cells. Taking all these data together, there is a strong inverse correlation between cellular levels of Hsp72 in SH-SY5Y cells and the susceptibility to apoptotic induction. Thus, applying Spearman's rank correlation analysis, *r* = −0.72, *p* = 0.03, results in a significant negative correlation between these two features. A reasonable conclusion from these correlations is that, in general, the susceptibility to STS-induced apoptosis in SH-SY5Y cells is determined by the cellular abundance of Hsp72.

**Table 1:** Correlation of cellular levels of Hsp72 with susceptibility to STS-induced apoptosis.

Undifferentiated cells	Treatment			
PC	**−**	**+**	**−**	**+**
Hsp72-Vector	**−**	**−**	**+**	**+**
Hsp72 Level	1	4.3±0.1	4.5±0.1	8.9±0.4
Apoptotic Index	28±2.3	11±1.4	11±1.7	3.3±1.5

Neuron-like cells	Treatment			
PC	**−**	**+**	**−**	**+**
Hsp72-Vector	**−**	**−**	**+**	**+**
Hsp72 Level	3.6±0.3	9.8±0.4	4.5±0.2	5.7±0.3
Apoptotic Index	11±1.6	5.3±1.0	3.5±2.2	3.0±2.7

Data are shown separately for undifferentiated and neuron-like SH-SY5Y cells after the treatments indicated.

PC indicates thermal preconditioning.

Hsp72-Vector indicates ectopic expression of Hsp72 as stably transfected cells (5YHSP72.1).

Hsp72 Level indicates that measured by western immunoblotting analysis (Figure 2), normalized to undifferentiated untransfected SH-SY5Y cells (data are expressed as the ratio relative to that control).

Apoptotic Index is the percentage of cells displaying apoptotic nuclei after treatment with STS (50 nM) for 12 h.

## Discussion

### The anti-apoptotic function of Hsp72

We have shown in our studies using the SH-SY5Y cell model that upon differentiation, neuron-like SH-SY5Y cells express and induce increased levels of Hsp72 compared to their undifferentiated proliferative form. The results presented here have shown that, in general, the greater the cellular levels of Hsp72 in SH-SY5Y cells subjected to various manipulations, the less is the susceptibility of cells to apoptotic induction by STS.

We explicitly show here that the block in apoptosis engendered by Hsp72 was upstream of mitochondrial engagement in apoptotic signalling, encompassing Bax recruitment and activation, and subsequent redistribution of cyt c. Such findings on SH-SY5Y cells analyzed here are in conformity with those in other non-neuronal cells types, including mouse embryo fibroblasts, human acute lymphoblastic T-cells and COS-7 cells where Hsp72 was found to block apoptotic signalling upstream of mitochondria [12], [26], [27], [28]. The results are consistent with findings in other cell lines [29], [30] where thermal preconditioning blocked subsequent mitochondrial engagement in apoptotic signalling although, in contrast to the present work, explicit involvement of Hsp72 was not demonstrated. Here we have used a variety of configurations of SH-SY5Y cells to demonstrate the ability of Hsp72 to block mitochondrial participation in apoptosis using this neuronal cell system.

In Table 1 it can be seen that neuronally differentiated 5YHSP72.1 cells show little induction of Hsp72 after thermal preconditioning and the corresponding apoptotic indices (after STS treatment) are close to the lower limit of detection. Nonetheless, comparing 5YHSP72.1 cells, either undifferentiated or neuron-like (without thermal preconditioning), each display cellular levels of Hsp72 4.5-fold increased over the control undifferentiated SH-SY5Y cells, but with significantly different susceptibilities to apoptotic induction by STS (apoptotic indices 11 and 3.5, respectively; Table 1). Note that the neuron-like 5YHSP72.1 cells seem to have acquired resistance to apoptosis above that reasonably ascribed to Hsp72. Therefore, there may be factors other than Hsp72 that are evident in differentiated 5YHSP72.1 neuron-like cells that affect apoptotic susceptibility.

It is possible that the Hsp72 levels observed in thermally preconditioned neuron-like 5YHSP72.1 cells are sufficient to block STS-induced apoptosis. On the other hand, these neuron-like 5YHSP72.1 cells are highly susceptible to severe heat challenges at 48°C [11] as well as severe hypoxic stress with glucose deprivation (L. Cheng and P. Nagley, unpublished observations). These severe insults cause very rapid cell death, indicating a unregulated necrotic-type death [31]. Hence, the resistance to STS-induced apoptosis observed in neuron-like 5YHSP72.1 cells suggests that Hsp72 may exclusively block mitochondrial regulated apoptotic cell death, with little effect on unregulated necrosis.

Our data do not formally eliminate the possibility of other possible downstream signalling events that could be blocked by Hsp72. Potential upstream targets for Hsp72-modulated apoptotic signalling include prosurvival members of the Bcl-2 family, such as Mcl-1 [32], in which case Hsp72 could be considered as an activator. It is not yet clear if proapoptotic BH3-only proteins, required for activation of Bax or Bak on mitochondria (prior to permeabilization of the outer mitochondrial membrane that facilitates redistribution of cyt c), are targets for Hsp72. In this case Hsp72 would need to be an inhibitor, to arrest apoptotic signalling upstream of mitochondria. Other potential targets for Hsp72 include blockade of stress signalling, including JNK, p38MAPK and p53, all of which may impact on cells in the intrinsic apoptosis pathway [13], [33], [34]. Whilst the correlation between Hsp72 levels and resistance to apoptotic death is strong (Table 1), our data do not eliminate the possibility that there may be other cytoprotective factors at play.

Interestingly, the increased basal expression and induction of Hsp72 observed in neuron-like SH-SY5Y cells is contrary to the situation of rat pheochromocytoma PC-12 cells differentiated with nerve growth factor which not only showed reduced levels of Hsp72 but also failed to induce Hsp72 upon heat treatment at 42°C for 30 min and 1 h [35]. Presumably, the regulation of Hsp72 in these neural model cells differs from that in SH-SY5Y cells.

In further support of the notion that Hsp72 is involved in the protection against STS, thermally preconditioned undifferentiated SH-SY5Y and 5YHSP72.1 cells became highly susceptible to apoptosis in the presence of KNK437 and STS. KNK437 is a benzylidene lactam compound reported to inhibit thermotolerance by blocking the upregulation of inducible Hsp72 [36], [37], [38]. KNK437 has been found to be more effective at inhibiting the inducible Hsp72 protein compared to the broad spectrum heat shock inhibitor quercetin [36], [37]. It has been shown that KNK437 functions by blocking the induction of Hsp70 at the mRNA level [38]. Therefore, it is possible that KNK437 inhibits other heat shock proteins which involve the activation of heat shock factor 1. This drug has also been reported to inhibit expression of Hsp105 and Hsp40 [37]. Further details of how KNK437 functions needs to be investigated in future studies. Nonetheless, we have shown that at non-toxic concentrations, KNK437 effectively restricts inducible Hsp72 expression to control (intrinsic) levels in our cell model [11]. In spite of possible off-target effects, the data obtained using KNK437 are consistent with Hsp72 being an important factor in both neuronal heat resistance and protection against apoptosis.

### Roles of Hsp72 in pathological context

Hsp72 has been found to be induced in a variety of pathological states, including cerebral ischemia and neurodegenerative diseases, such as Alzheimer's disease [1], [39], [40]. In experimental mouse models, the expression of Hsp72 is upregulated in the brain several hours after cerebral insult and has been correlated with better recovery [6], [41]. In cases of a severe ischemic attack, there is a relatively small induction of Hsp72 in the affected area. Hence, the outcome and amount of injury to the area seems to correlate with the expression of Hsp72 detected in the region. Preconditioning has also been used widely in several studies to induce Hsp72 and protect against a subsequent hypoxic insult [30], [42], [43].

In Alzheimer's disease, where the accumulation of misfolded proteins is the major cause of this neurodegenerative disorder, the increased expression of Hsp72 rescues neurons from the toxic effects of intracellular beta-amyloid 42 (Abeta42) accumulations [44], [45]. Moreover, in an ALS model, Hsp72 upregulation protects NSC34 cells against apoptosis associated with the presence of mutant SOD1 aggregates, yet does not affect the frequency of aggregate formation (K.Y Soo and P. Nagley, unpublished observations). An understanding of the interplay between Hsp72 levels and cell survival demonstrated in this study, may lead to new strategies for treatment and drug design targeting neuronal survival. Compounds that can up-regulate Hsp72 levels can potentially be used in the treatment of various cerebral insults and neurodegenerative disorders to suppress excessive cell death and allow neuronal function to be retained.

### Conclusion

This study indicates that Hsp72 has a key role in protection against apoptosis and that it acts upstream of Bax activation. The data obtained indicate that the degree of protection to apoptotic stress is dependent not only on the basal levels of Hsp72 but also on the amount of Hsp72 induced in the various cellular scenarios analyzed. This study supports the idea that if neurons express beneficial levels of Hsp72 or are exposed to a preconditioning treatment to induce Hsp72, they acquire neuroprotective features that provide these post-mitotic cells with the ability to withstand various stresses, thus enhancing their survival.

## Materials and Methods

### Reagents

Primary antibodies used for immunocytochemistry were rabbit polyclonal Hsp60 from Abcam (Cambridge, USA); mouse monoclonal Bax (clone 6A7) and mouse monoclonal cyt c (clone 6H2.B4) from BD Biosciences Pharmingen (San Jose, USA). Fluorescent secondary antibodies for immunocytochemistry conjugated to Alexa 488 or Alexa 568 were purchased from Molecular Probes (Eugene, USA). Staurosporine (Sigma, USA) was dissolved in dimethyl sulfoxide (DMSO) to a final stock concentration of 500 µM. KNK437 (N-formyl-3,4-methylenedioxy-benzylidene-γ-butyrolactam, EMD Biosciences Inc, USA) was titrated to the appropriate concentration for experimental use as previously described [11]. Note that for various concentrations of KNK437 and STS applied, the input of DMSO was held constant at 0.1%.

### Cell culture

Human neuroblastoma SH-SY5Y cells (ATCC catalogue number CRL-2266) were cultured in DMEM medium (Gibco, USA) supplemented with 1 mM L-Glutamine, 10 mM HEPES and 10% heat inactivated foetal calf serum (CSL Limited, Australia) as described [11]. SH-SY5Y cells stably expressing exogenous Hsp72 (denoted 5YHSP72.1) were generated by transfecting pCI-neo Hsp72 [46] using the transfection reagent LipofectAMINE PLUS (Invitrogen, USA) according to manufacturer instructions. Stably transfectant clones were selected and maintained in medium containing G418 (500 µg/ml). Procedures for neuronal differentiation were carried out as described [11]. Briefly, to differentiate SH-SY5Y and 5YHSP72.1 cells, undifferentiated cells were treated with 10 µM of *all-trans* retinoic acid (Sigma-Aldrich, USA) in DMEM. After 5 days, RA and was removed and 5 ng/ml of BDNF (Sigma-Aldrich, USA) was added to the cells in DMEM without serum for a further 2 days. Thermal preconditioning was performed by immersing cells in a water bath (Memmert, Germany) at 43°C (±0.1°C) for 30 min and then allowed to recover at 37°C for 8 h. Control cells were maintained at 37°C throughout.

### Cell Titer-Blue assays

Cells were seeded in a 24-well tissue culture plate and treated with STS. At the end of the test exposure period, 20 µl Cell Titer-Blue reagent (Promega, USA) was added per 100 µl media and mixed gently for 10 sec. The cells were incubated at 37°C in the absence of light for a sufficient time (usually 2 to 3 h) for detectable reduction of resazurin to resorufin in the untreated cell control samples, determined by the color change from blue to pink. Fluorescence was immediately read with 560 nm excitation and 590 nm emission wavelengths using an automated microplate-based multi-detection reader (BMG Fluorostar Optima; BMG Labtech, Germany). The mean fluorescence value from control reaction mixtures with culture medium (lacking cells) was subtracted from fluorescence values of experimental wells.

### Western immunoblotting and densitometry analysis

Western immunoblotting was carried out as previously described [11]. Briefly, cells were collected and resuspended in cell lysis buffer (1% Triton X-100, 4 mM EGTA, 15 mM MgSO_4_, 25 mM glycylglycine) for 5 min on ice. The suspension was centrifuged at 10, 000× g for 5 min and the supernatant was collected. The protein concentration of each sample was determined using a protein assay kit (BCA protein assay, Pierce, USA). Cell lysates containing equivalent amounts of protein were resolved on 12% SDS-PAGE gel, transferred to PVDF membrane and probed with antibodies specific for Hsp72 (SPA 810, Stressgen Biotechnologies, USA) and actin (Neomarkers, USA). Bands were detected by the use of secondary antibodies conjugated to horseradish peroxidase (GE Healthcare, Australia). The PVDF membrane was then treated with enhanced chemilumiescence substrate (Bio-Rad Laboratories, Australia) for 5 min. The PDVF membrane was then exposed to hyperfilm (GE Healthcare, Australia) to visualise the bands. The film was then scanned and band density values were analyzed using the ImageQuant TL densitometry program (GE Healthcare, UK).

### Fixing and staining cells for imaging

Following treatment of undifferentiated and neuron-like cells with STS, adherent cells (mostly viable) were trypsinized and combined with detached cells (dead or dying) to recover the total cell population for each sample. The cells were then centrifuged in a cytospin centrifuge on coverslips coated with poly-D-lysine (Sigma-Aldrich, USA) to collect adherent and floating cells. Cells were immediately fixed with 4% (w/v) paraformaldehyde (Electron Microscopy Sciences, USA) for 15 min at 37°C and permeabilized with 0.1% Triton-X-100 (BDH Chemicals, USA) for 10 min at room temperature. In experiments on Bax activation, cells were permeabilized with CHAPS buffer (150 mM NaCl, 10 mM HEPES, 1.0% CHAPS) for 10 min at room temperature. The cells were blocked with 3.5% bovine serum albumin (BSA) and incubated at room temperature for a minimum of 30 min before immunostaining with the relevant primary antibody, overnight at 4°C. The cells were rinsed three times with phosphate-buffered saline (PBS) and exposed to the relevant secondary antibody for 1 h at room temperature. Cells were rinsed three times with PBS to remove excess secondary antibody and then stained with 0.5 µg/ml DAPI for 10 min, followed by a final rinse with PBS. The coverslips were inverted and mounted on glass slides using permafluor (Thermo Scientific, USA) ready for microscopic visualization by confocal laser scanning microscopy.

### Confocal laser scanning microscopy

Cells were imaged using an Olympus Fluoview 500 confocal laser scanning microscope, equipped with a HeCd/Argon/HeNe laser light source with peak emissions at 442, 488 and 543 nm. Images were acquired using a 60× water immersion objective lens. Photomultiplier tube levels were set to a level at which bleed-through effects from one channel to the other were negligible in untreated control cells. The same photomultiplier tube settings were then applied for their STS-treated counterpart cells. The computer software ImageJ (National Institute of Health, USA) was used to process images acquired from the confocal laser scanning microscope.

### Statistical tests

For analysis of quantified data for apoptotic indicators, for each treatment, all cells within 3–4 microscopy fields were scored until a threshold of 300 cells was reached, after which all remaining cells in the final field were scored. Results were obtained from at least three independent experiments and expressed as mean ± standard deviation. Student's t-test was applied for appropriate comparisons. Correlations between susceptibility to STS-induced apoptosis and cellular Hsp72 levels were analyzed using the Spearman's rank correlation coefficient (*r*). Calculations for statistical tests were made using Microsoft Excel software package. For both tests, a probability level (*p*)<0.05 was considered significant.
